# Assessment of Some Unsymmetrical Porphyrins as Promising Molecules for Photodynamic Therapy of Cutaneous Disorders

**DOI:** 10.3390/ph17010062

**Published:** 2023-12-29

**Authors:** Andreea Mihaela Burloiu, Gina Manda, Dumitru Lupuliasa, Radu Petre Socoteanu, Dragos Paul Mihai, Ionela Victoria Neagoe, Laurentiu-Iliuta Anghelache, Mihaela Surcel, Mihai Anastasescu, Laura Olariu, Cerasela Elena Gîrd, Stefania Felicia Barbuceanu, Luis Filipe Vieira Ferreira, Rica Boscencu

**Affiliations:** 1 Faculty of Pharmacy, “Carol Davila” University of Medicine and Pharmacy, 6 Traian Vuia St., 020956 Bucharest, Romania; andreea-mihaela.burloiu@drd.umfcd.ro (A.M.B.); dumitru.lupuliasa@umfcd.ro (D.L.); dragos_mihai@umfcd.ro (D.P.M.); cerasela.gird@umfcd.ro (C.E.G.); stefania.barbuceanu@umfcd.ro (S.F.B.); 2 “Victor Babeş” National Institute of Pathology, 050096 Bucharest, Romania; ionela.neagoe@ivb.ro (I.V.N.); laurentiu.anghelache@ivb.ro (L.-I.A.); mihaela.surcel@ivb.ro (M.S.); 3 “Ilie Murgulescu” Institute of Physical Chemistry, Romanian Academy, 060021 Bucharest, Romania; psradu@yahoo.com (R.P.S.); manastasescu@icf.ro (M.A.); 4 “SC. Biotehnos SA”, 3-5 Gorunului St., 075100 Bucharest, Romania; lolariu@biotehnos.com; 5 BSIRG—Biospectroscopy and Interfaces Research Group, iBB-Institute for Bioengineering and Biosciences, Instituto Superior Técnico, Universidade de Lisboa, 1049-001 Lisboa, Portugal; lfvieiraferreira@tecnico.ulisboa.pt

**Keywords:** unsymmetrical porphyrin, photodynamic therapy, HaCaT keratinocytes, Hs27 skin fibroblasts, SCL II skin squamous cell carcinoma, B16F10 melanoma cells, atomic force microscopy

## Abstract

In order to select for further development novel photosensitizers for photodynamic therapy in cutaneous disorders, three unsymmetrical porphyrins, namely 5-(4-hydroxy-3-methoxyphenyl)-10,15,20-tris-(4-acetoxy-3-methoxyphenyl) porphyrin (P2.2), 5-(2-hydroxy-5-methoxyphenyl)-10,15,20-tris-(4-carboxymethylphenyl) porphyrin (P3.2), and 5-(2,4-dihydroxyphenyl)-10,15,20-tris-(4-acetoxy-3-methoxyphenyl) porphyrin (P4.2), along with their fully symmetrical counterparts 5,10,15,20-tetrakis-(4-acetoxy-3-methoxyphenyl) porphyrin (P2.1) and 5,10,15,20-tetrakis-(4-carboxymethylphenyl) porphyrin (P3.1) were comparatively evaluated. The absorption and fluorescence properties, as well as atomic force microscopy measurements were performed to evaluate the photophysical characteristics as well as morphological and textural properties of the mentioned porphyrins. The cellular uptake of compounds and the effect of photodynamic therapy on the viability, proliferation, and necrosis of human HaCaT keratinocytes, human Hs27 skin fibroblasts, human skin SCL II squamous cell carcinoma, and B16F10 melanoma cells were assessed in vitro, in correlation with the structural and photophysical properties of the investigated porphyrins, and with the predictions regarding diffusion through cell membranes and ADMET properties. All samples were found to be isotropic and self-similar, with slightly different degrees of aggregability, had a relatively low predicted toxicity (class V), and a predicted long half-life after systemic administration. The in vitro study performed on non-malignant and malignant skin-relevant cells highlighted that the asymmetric P2.2 porphyrin qualified among the five investigated porphyrins to be a promising photosensitizer candidate for PDT in skin disorders. P2.2 was shown to accumulate well within cells, and induced by PDT a massive decrease in the number of metabolically active skin cells, partly due to cell death by necrosis. P2.2 had in this respect a better behavior than the symmetric P.2.1 compound and the related asymmetric compound P4.2. The strong action of P2.2-mediated PDT on normal skin cells might be an important drawback for further development of this compound. Meanwhile, the P3.1 and P3.2 compounds were not able to accumulate well in skin cells, and did not elicit significant PDT in vitro. Taken together, our experiments suggest that P2.2 can be a promising candidate for the development of novel photosensitizers for PDT in skin disorders.

## 1. Introduction

Porphyrins represent a class of tetrapyrrolic compounds known for their role in a wide range of clinical applications with great impact on the health state of the population [1,2,3,4,5,6,7,8]. From a multidisciplinary research point of view, these tetrapyrroles types have a vast potential for function as building blocks in diagnosis and therapeutic applications, especially in the oncological field but also in photodynamic therapy to eradicate of bacteria, fungi, and viruses [1,2,3,4,5,6,7,8]. Recently, porphyrin-type derivatives proved to be valuable therapeutic agents in the treatment by PDT of various skin disorders such actinic keratosis, cutaneous infections, and inflammatory dermatoses, as well as in cutaneous T-cell lymphoma [9].

Photodynamic therapy (PDT) is based on a provoked burst of singlet oxygen that is highly efficient in killing cells [10]. In the treatment of skin disorders, PDT consists in topical administration of a photosensitizer (PS), followed by PS activation induced by precise tissue illumination with harmless visible light of well-defined wavelength, for generating a localized singlet oxygen burst in the presence of molecular oxygen, hence inflicting oxidative damage to the illuminated tissue. PDT is a highly targeted therapeutic approach because PS is only locally activated by laser irradiation in the diseased tissue. PDT with topically applied PS offers the advantage of field PDT, hence decreasing the risk of systemic phototoxicity [9,10,11]. Actinic keratosis is a premalignant lesion occurring on chronically sun-damaged skin, characterized by neoplastic proliferation of keratinocytes in the epidermis. Topical PDT using aminolevulinic acid (ALA) or its methyl ester proved to be a frequent option for AK treatment [12], but the clinical potential of ALA is limited by the low rate of its uptake in cells and its poor bioavailability. The main disadvantage of ALA is its hydrophilicity that accounts for its relatively low ability to penetrate the skin. In addition, some patients have reported pain when using ALA as PS in PDT for skin disorders [13]. In addition to ALA, some porphyrin derivatives, such as Verteporfin and Hematoporphyrin, started to be used for PDT-mediated keratolytic treatment, having superior efficacy as compared to ALA [14,15].

Porphyrin derivatives are versatile structures, with molecular architectures that can be tailored by attaching substituents with various degrees of polarity for an optimal and fit-to-purpose hydrophilic/lipophilic ratio that can significantly increase their accumulation in the diseased tissue. A major advantage of porphyrins is their selectivity for tumor cells and, in contrast to ALA, they do not need metabolization for becoming PS, their tetrapyrrolic structure is stable, and they have good photodynamic properties for PDT [16]. Through their structural profile and electronic load, porphyrins can efficiently generate reactive oxygen species (ROS), such as singlet oxygen, in the presence of molecular oxygen and light [5,6,17,18,19,20]. A series of pharmaceutical products containing porphyrin-type photosensitizers (e.g., Metvix^®^, Levulan^®^, Photogem^®^, Purlytin^®^, Foscan^®^, Foslip^®^), are presently used for various skin disorders. The main disadvantages reported for these photosensitizers were related to the solubility in biological fluids and molecular aggregation tendency, with a negative effect on their uptake in tumor cells [21,22,23,24]. These disadvantages can be limited by implementing drug design strategies for obtaining new porphyrin-type PS with a superior balance of their hydrophilic/hydrophobic properties that favors an efficient cellular uptake. A series of A3B or A2B2-type unsymmetrical porphyrins, with an acceptable volume of functional groups (−OH, −OCOCH_3_ and −COCH_3_), was obtained by our research group using ecological and versatile approaches [25,26,27,28,29,30,31,32].

With the purpose to select novel porphyrins as photosensitizers for PDT in skin disorders, three unsymmetrical porphyrins, namely 5-(4-hydroxy-3-methoxyphenyl)-10,15,20-tris-(4-acetoxy-3-methoxyphenyl) porphyrin (P2.2), 5-(2-hydroxy-5-methoxyphenyl)-10,15,20-tris-(4-carboxymethylphenyl) porphyrin (P3.2), and 5-(2,4-dihydroxyphenyl)-10,15,20-tris-(4-acetoxy-3-methoxyphenyl) porphyrin (P4.2), along with the corresponding symmetrical structures, 5,10,15,20-tetrakis-(4-acetoxy-3-methoxyphenyl) porphyrin (P2.1) and 5,10,15,20-tetrakis-(4-carboxymethylphenyl) porphyrin (P3.1) were investigated.

Their photophysical properties were evaluated, mainly regarding spectral behavior, while their aggregation tendency was studied by atomic force microscopy. The interaction of porphyrins with biologic membranes, and the ADMET properties were predicted through computational studies. Porphyrins were further investigated in vitro on human skin-relevant cell lines, either normal (HaCaT keratinocytes and Hs27 fibroblasts) or tumoral (SCL II squamous cell carcinoma), as well as on mouse B16F10 melanoma cells.

## 2. Results and Discussion

### 2.1. Photophysical Characterization of the Investigated Porphyrins

#### 2.1.1. Absorption Spectra

The particular spectral characteristics associated with porphyrinic structures recommend this structural type in PDT applied to malignant diseases [1,5,17,18,19,20,33]. The molecular absorption spectrum of a porphyrin is described by five spectral bands: an intense band (Soret band) in the 390–440 nm spectral range and four other Q bands located in the 440–700 nm spectral range [34,35,36]. Although the absorption intensity is much higher in the Soret band domain, the spectral range of interest is that of the Q bands from the point of view of PDT in which the porphyrin photosensitizer is activated with laser light (580–700 nm). In addition, the molecular structure, respectively, the aromatic character imprinted on the molecule by the π electron system in the tetrapyrrolic nucleus, endows porphyrins with the ability to emit fluorescence in the 600–800 nm spectral range, hence making them imagistic agents. By irradiation with red light and in the presence of molecular oxygen, these structures generate massively cytotoxic ROS, more specifically singlet oxygen.

The photophysical potential of porphyrinic chromophores is directly influenced by the nature of the substituents in the meso positions of the tetrapyrrolic nucleus, through the induced electronic effects on the π electron system, with consequences on this type [34,35,36,37].

**Figure 1:**
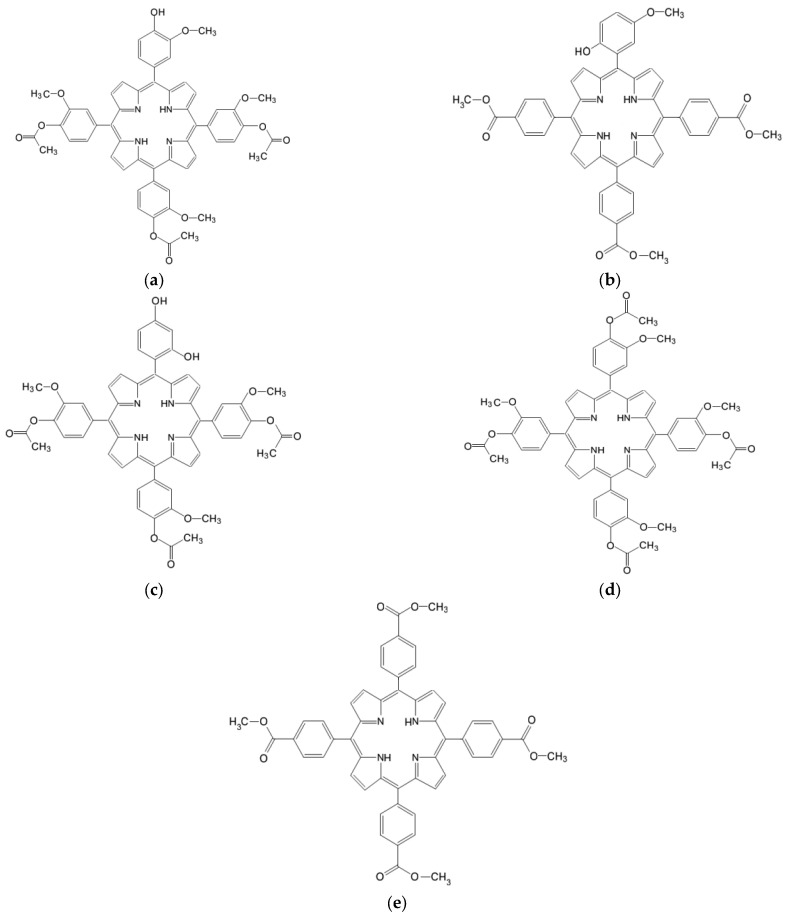
The molecular structure of the investigated porphyrins: (**a**) 5-(4-hydroxy-3-methoxyphenyl)-10,15,20-tris-(4-acetoxy-3-methoxyphenyl) porphyrin (**P2.2**), (**b**) 5-(2-hydroxy-5-methoxyphenyl)-10,15,20-tris-(4-carboxymethylphenyl) porphyrin (**P3.2**), (**c**) 5-(2,4-dihydroxyphenyl)-10,15,20–tris-(4-acetoxy-3-methoxyphenyl) porphyrin (**P4.2**), (**d**) 5,10,15,20-tetrakis-(4-acetoxy-3-methoxyphenyl) porphyrin (**P2.1**), (**e**) 5,10,15,20-tetrakis-(4-carboxymethylphenyl) porphyrin (**P3.1**).

Therefore, this research was on the design and selection of new synthetic porphyrin compounds (Figure 1) with photosensitizing potential for the therapy of premalignant and malignant skin conditions, included the photophysical evaluation of porphyrins, dissolved in polyethylene glycol (PEG) 200, either undiluted or diluted in phosphate buffer solution, at the final concentration of 10 μM porphyrin.

**Figure 2:**
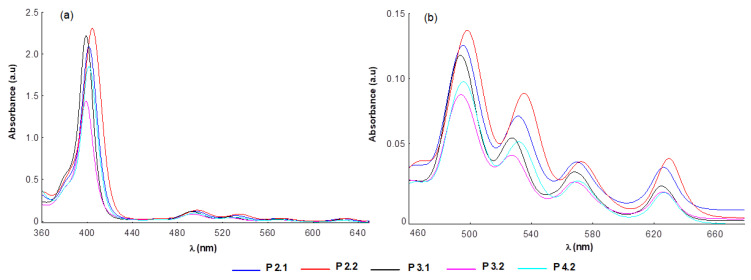
Absorption spectra of porphyrins (10 μM) dissolved in PEG 200 (**a**) and magnification of the corresponding Q bands region (**b**).

**Figure 3:**
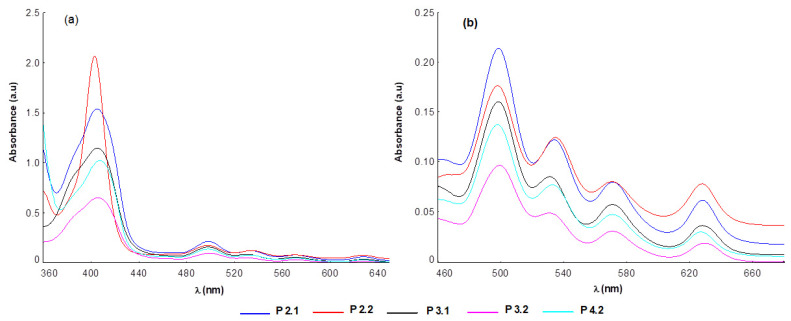
Absorption spectra of porphyrins (10 μM) in PEG 200/PBS (1/1000) (**a**) and magnification of the corresponding Q bands region (**b**).

Table 1 shows the spectral parameters associated with the absorption and emission spectra of the porphyrins included in this study. At the concentration of 10 μM porphyrin, the molecular absorption spectra of the compounds P2.1, P2.2, P3.1, P3.2, and P4.2 had the typical profile of porphyrinic compounds, and did not indicate molecular association phenomena (Figure 2 and Figure 3). In the spectral range 400–407 nm, the Soret band was highlighted as a result of an intense absorption, and the Q bands were identified in the spectral region of 495–629 nm. The presence of the absorption maximum associated with the band Qx(0,0) at 621–630 nm confirmed the fact that the investigated porphyrin compounds absorbed in the phototherapeutic range [19,20].

Small changes in the positioning of the absorption maxima were evidenced (Figure 2 and Figure 3) as an effect of the structural differences between the tested compounds. Thus, the spectral bands of the asymmetric compounds were slightly shifted towards the red compared to those of the symmetric structures in the same solvent.

Regarding the intensity of the absorption bands, the highest values of the molar extinction coefficients were associated with compound P2.2, and the smallest with porphyrin P3.2 (Figure 2 and Figure 3). Compared to the spectral region of therapeutic interest (621–630 nm), the best absorptions were registered for the P2.2 and P2.1 compounds (Figure 2 and Figure 3). According to published data [34,35,36], the changes found in the intensities and positions of the spectral bands are the consequence of: (i) the different structure of the functional groups attached to the porphyrinic macrocycle, and (ii) the energy values that accompany the electronic transitions between the orbitals fully occupied with electrons (a2u and a1u) and the unoccupied orbitals (e1g).

Due to the nature of the peripheral substituents of the tetrapyrrole macrocycle, the P2.1, P2.2, and P4.2 compounds had a higher electronic charge compared to P3.1 and P3.2. Conjugation effects may appear between the electrons of these substituents and the electrons of the a2u orbitals, leading to an increased absorption intensity (Figure 2 and Figure 3). In turn, the presence of two –OH functional groups in the structure of P4.2 increased the potential for hydrogen bonds formation with the solvent molecules, consequently decreasing the absorption intensity as compared to P2.2 and P2.1.

The spectral profile of the investigated porphyrins did not change significantly in solutions with a content of 10 μM porphyrin in PEG 200/PBS (1/1000), as compared to porphyrin solutions in PEG 200 (Table 1). Nevertheless, a slight increase in the absorption intensity, accompanied by small bathochromic shifts of the spectral bands, was highlighted (Figure 3).

#### 2.1.2. Emission Spectra

**Figure 4:**
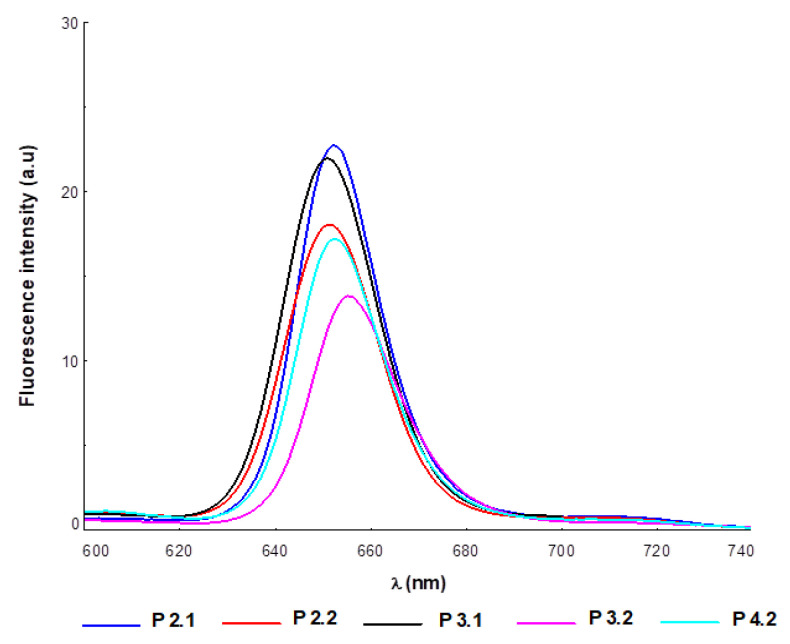
Emission spectra of porphyrins at a concentration of 10 μM in PEG 200 as solvent; λex = 410 nm.

The emission properties of the investigated compounds were influenced by the nature of the solvent and the electronic effects dictated by the structural particularities of each porphyrin. For the same solvent, the emission spectra of the investigated porphyrins kept the spectral profile typical of the fluorescence of free base porphyrins, with a decrease in fluorescence in the order P2.1 > P3.1 > P2.2 > P4.2 > P3.2 (Figure 4).

Regarding the fluorescent properties of the investigated compounds at a concentration of 10 μM porphyrin in PEG 200/PBS (1/1000), small shifts of the spectral bands were observed, with a decrease in fluorescence intensity (more for P3.1, P2.2, and P4.2) compared to porphyrin solutions of the same concentration in PEG 200 (Table 1). These spectral changes might be due to the formation of molecular associations through physical interactions when using the PEG 200/PBS solution as solvent, which produces fluorescence extinction. Regardless of the nature of the solvent, the symmetric structures P2.1 and P3.1 exhibited higher fluorescence than the asymmetric ones (P2.2, P4.2, P3.2) (Table 1). The decrease in the fluorescent signal was a consequence of the electronic effects induced by the –OH functional group, and of displacements of the electron density in the π-conjugated systems of the porphyrinic structure. As an example, the P2.2 and P4.2 asymmetric structures had weaker fluorescence as compared to the symmetrical structure P2.1.

Concluding, under the experimental conditions addressed, the investigated compounds confirmed absorption in the expected range relevant for PDT. Furthermore, emission spectra had the typical profile of the spectrum associated with a porphyrinic photosensitizer, with small shifts of the emission maxima in the case of the porphyrin solutions in PEG 200/PBS (1/1000). The compounds P2.1, P3.1, and, to a lesser extent, P2.2 exhibited good fluorescence, hence being promising imaging agents.

### 2.2. Aggregation Study on Porphyrinic Structures

**Figure 5:**
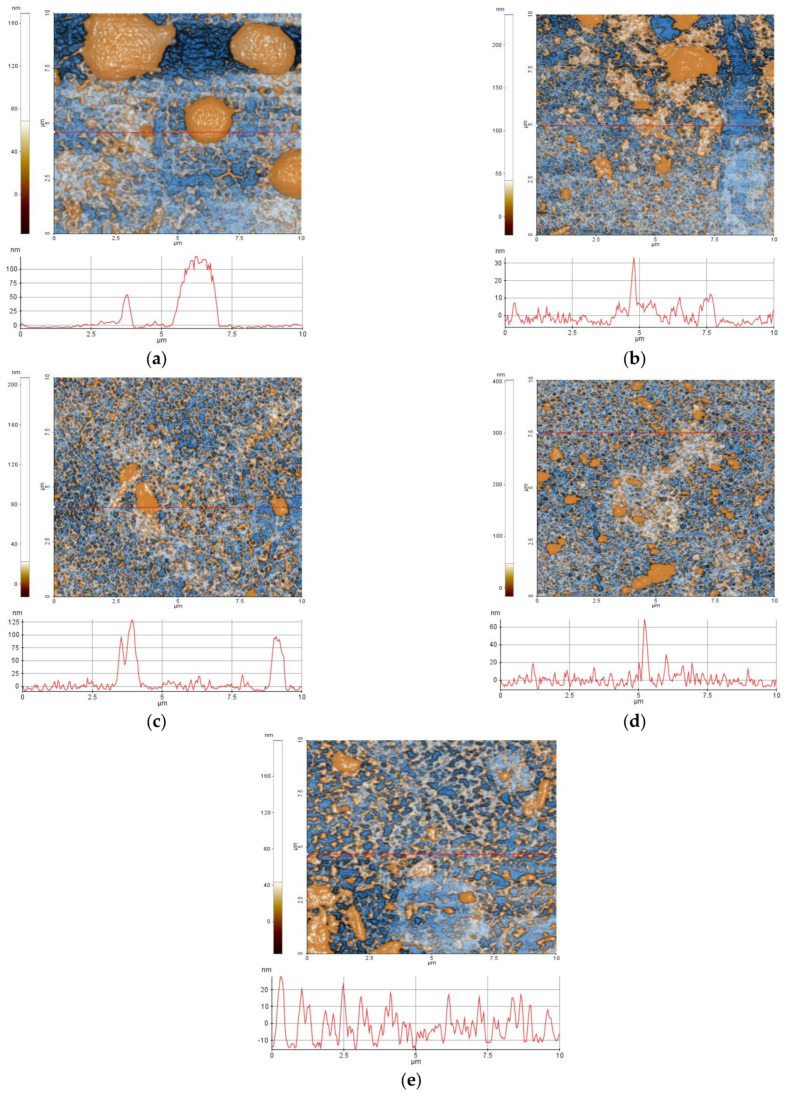
Enhanced contrast bi-dimensional (2D) topographic AFM images of samples P2.1 (**a**), P2.2 (**b**), P3.1 (**c**), P3.2 (**d**), and P4.2 (**e**), scanned over an area of (10 × 10) μm^2^, together with random cross-sectional height examples (line scans).

Enhanced-color bi-dimensional (2D) topographic AFM images of the samples P2.1, P2.2, P3.1, P3.2, and P4.2 in PEG 200, scanned over an area of (10 × 10) μm^2^, were obtained, together with characteristic surface profiles (line scans) (Figure 5). All samples exhibited an alternation of smooth areas and some aggregation of the material. Smaller particles (aggregates) started from tens of nm and ended up in hundreds of nm in diameter. The aggregation of porphyrins may be due to the adherence to the substrate used for deposition (microscopic glass substrate) but also to the nature of the dispersion medium (PEG 200). Based on AFM investigations at the scale of (10 × 10) μm^2^, it was found that compound P2.1 had the highest aggregation tendency (Figure 5a).

**Figure 6:**
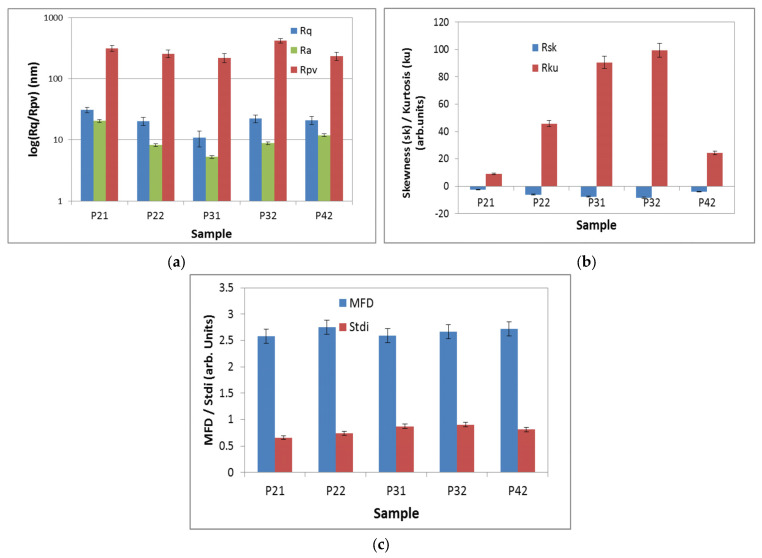
The corrugation and textural parameters at the scale of (10 × 10) μm^2^: RMS and Ra roughness and peak-to-valley parameter (**a**); Skewness and kurtosis (**b**), Mean fractal dimension (MFD) and textural index ratio—Stdi (**c**).

The corrugation parameters (RMS—(Rq) and average (Ra) roughness as well as peak-to-valley (Rpv) parameters), as estimated at the scale of (10 × 10) μm^2^, were plotted (Figure 6a). The logarithmic scale was used due to the high difference between Rq/Ra and Rpv. Similar values were observed in RMS roughness for the investigated set of porphyins, the smallest being registered for sample P3.1 (Rq = 10.8 nm) and the highest for P2.1 (Rq = 30.8 nm). The Rpv values were in the range of 220–320 nm. The slightly negative values of skewness (Rsk) might be regarded as a “porosity”-like morphology of the porphyrin layers on glass, while the sharp values of the kurtosis (Rku) as a Cauchy distribution of the surface events (asymmetric distribution with heavy tails) (Figure 6b). All samples were isotropic (Stdi > 0.5) and self-similar, with MFD values over 2.5 (rough surfaces) (Figure 6c).

**Figure 7:**
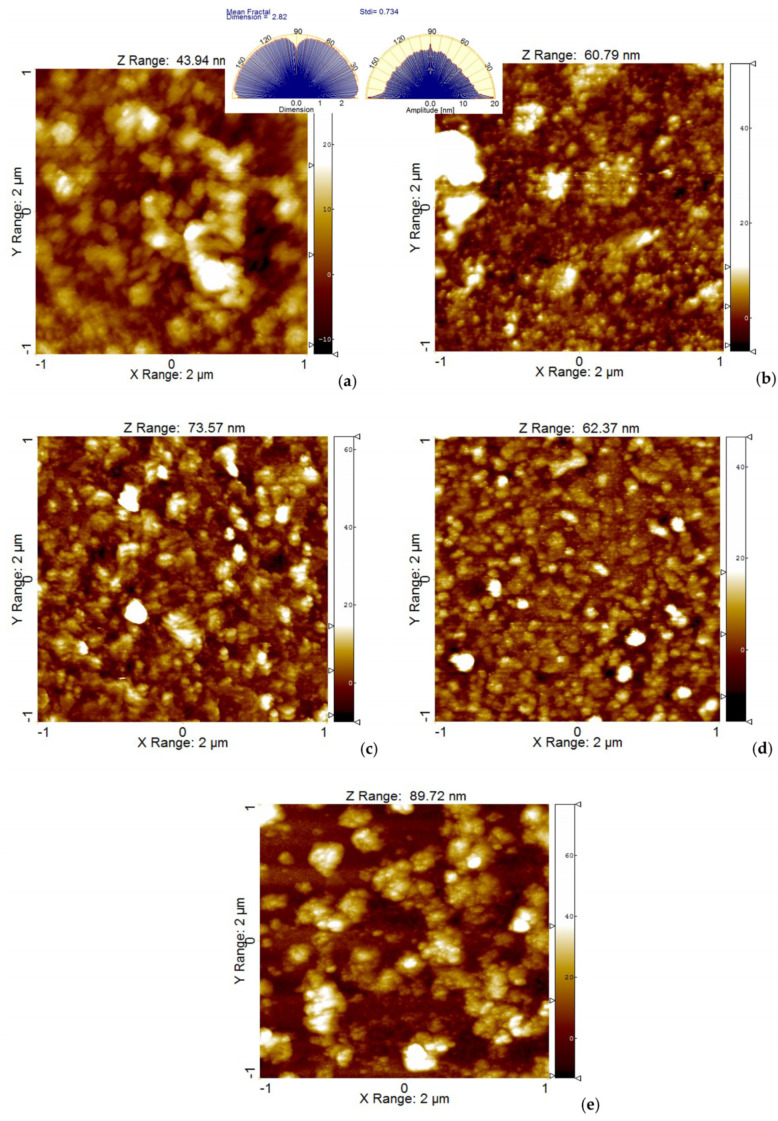
Bi-dimensional (2D) topographic AFM images of samples P2.1 (**a**), P2.2 (**b**), P3.1 (**c**), P3.2 (**d**), and P4.2 (**e**), scanned over an area of (2 × 2) μm^2^. Spectra obtained from AFM images (calculated at different angles by Fourier amplitude spectrum) for Mean fractal dimension—Sample P2.1, and amplitude—Sample P2.2, are inserted in the corresponding AFM images (**a**,**b**) for exemplification.

**Figure 8:**
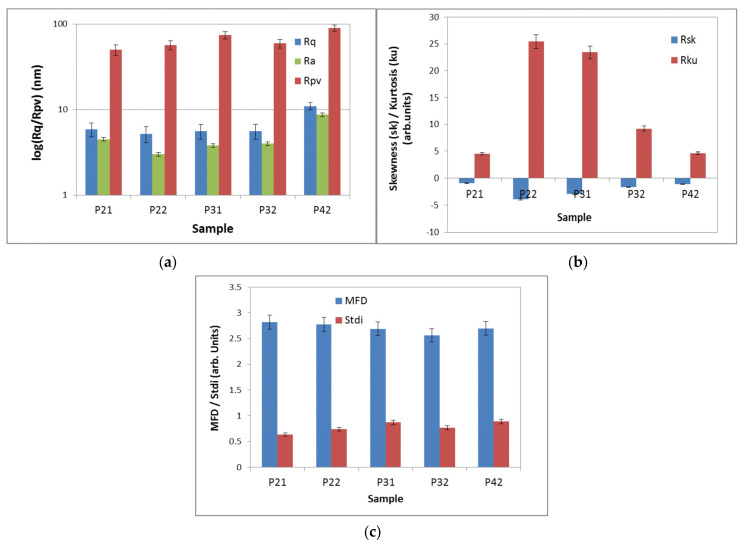
The corrugation and textural parameters at the scale of (2 × 2) μm^2^: RMS and Ra roughness and peak-to-valley parameter (**a**); Skewness and kurtosis (**b**), Mean fractal dimension (MFD) and textural index ratio—Stdi (**c**).

The samples were further investigated at a lower scale of (2 × 2) μm^2^, outside of large defects created by the aggregation of materials, and bi-dimensional (2D) topographic AFM images of the samples P2.1, P2.2, P3.1, P3.2, and P4.2, presented as classical colored images (single gradient z-scale), were obtained (Figure 7). In this case, the samples were locally smoother (less than 10 nm roughness), having typical Rpv values of 50–60 nm (Figure 7 and Figure 8), and the height distribution histograms were less sharp compared to the larger scales (Figure 6b), except for samples P2.2 and P3.2, which still exhibited some protruding local particles. The self-similarity nature of the investigated surfaces, evaluated from the fractal behavior, and the isotropic character of the coatings formed by the dispersion of porphyrins in PEG 200 were maintained, being scale-independent.

### 2.3. In Silico Approaches

#### 2.3.1. Prediction of the Diffusion of Porphyrin Structures through the Cell Membrane

Since the cellular uptake of porphyrin derivatives is essential for acting as effective photosensitizers in cells and tissues, we first predicted the translocation of the investigated compounds through the cell membrane using the PerMM web-server.

**Figure 9:**
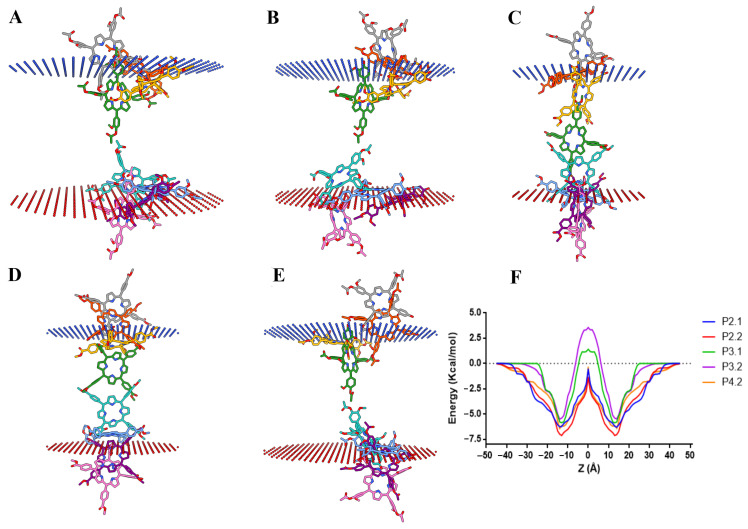
(**A**–**E**) Representative snapshots illustrating the predicted translocation pathways across the lipid bilayer for P2.1 (**A**), P2.2 (**B**), P3.1 (**C**), P3.2 (**D**), and P4.2 (**E**); (**F**) Variation of the transfer energy (ΔG_transf_) as a function of distance from the center of the lipid bilayer (Z).

The membrane transfers energy (ΔG_transf_) profiles along with the lipid bilayer normal (Z) and the optimized spatial conformations for the investigated porphyrin derivatives are illustrated in Figure 9, while the predicted membrane binding energies (ΔG) and the calculated permeability coefficients (logPerm) are shown in Table 2.

The lowest binding energy was predicted for P2.2, followed by P4.2, P2.1, P3.1, and P3.2, indicating that two out of the three investigated asymmetric porphyrins (P2.2 and P4.2) and one symmetrical porphyrin (P2.1) have high estimated affinities for membrane lipids. Similarly, P2.2 showed the highest calculated permeation coefficient (LogPerm), while P3.1 and P3.2 had the lowest values. Interestingly, the porphyrin derivatives P4.2 and P2.1 had relatively similar LogPerm values. Even though P4.2 showed a slightly higher affinity for the lipid membrane, P2.1 required less transfer energy to diffuse through water molecules and to bind at the membrane–water interface, as seen in the ΔG_transf_(Z) profile (Figure 9F, ±15–30 Å from bilayer center). For all compounds, the transbilayer energy profiles had an energy minimum at the lipid–water interface and a maximum corresponding to the membrane center. The energy profiles across the hydrophobic portion of the membrane (Figure 9F, ±15 Å from bilayer center) were similar for P2.1, P2.2, and P4.2, showing almost superposable shapes for P2.2 and P4.2, suggesting that there are no significant differences with respect to the difficulty of crossing the lipid acyl chain region. On the other hand, compounds P3.1 and P3.2 required high, positive transfer energies to permeate across the membrane core, higher energy values being also registered at the water–lipid interface. Therefore, the porphyrin compounds P2.2, P4.2, and P2.1 were highly likely to permeate the cell membrane according to the calculated LogPerm values and the ΔG_transf_(Z) profiles, while the translocation of P3.1 and P3.2 could be achieved with increased difficulty, requiring higher transfer energies, although P3.2 had the potential to interact with the hydrophilic heads of membrane lipids through hydrogen bonding.

#### 2.3.2. Prediction of ADMET Properties

Several pharmacokinetic and toxicological properties were predicted for the investigated compounds using three web-servers. Considering that the porphyrin derivatives were designed for topical administration in skin disorders, the most relevant ADMET properties are skin permeability and skin sensitization. However, many active substances can permeate through the skin to the subcutaneous tissue following local application, and are thereafter absorbed in the circulatory system, leading to undesirable systemic toxicity. Predicted pharmacokinetic properties are shown in Table 3, while toxicity parameters are presented in Table 4.

All the porphyrin derivatives had the same value for the predicted logarithmic skin permeability (log Kp, −2.735 cm/h), which falls within the acceptable range for drugs [38]. If we consider oral administration, all compounds showed positive predicted intestinal absorption, while only P3.2 had high predicted oral bioavailability. All compounds were predicted as potential P-glycoprotein inhibitors. Moreover, P4.2 was predicted as a P-glycoprotein substrate, while P2.1, P2.2, and P3.1 were estimated as either substrates or non-substrates, the results being inconclusive. All the investigated compounds were predicted as potential hepatic transporters OATP1B1 and OATP1B3 inhibitors, but only P2.2 and P4.2 were identified as potential OATP2B1 inhibitors. The predicted subcellular localization was the mitochondrion, which could improve the efficacy of PDT. The prediction of interactions with cytochrome P450 isoforms was inconclusive since the evaluated compounds were predicted as both inhibitors and non-inhibitors by the ADMET algorithms. For instance, none of the compounds were predicted as CYP2D6 substrates or inhibitors, but all five compounds were shown to have the potential to be either substrates or inhibitors of CYP1A2, CYP2C9, CYP2C19, and CYP3A4, and were positive for CYP inhibition promiscuity. All compounds had predicted plasma protein binding higher than 80%, the highest values being noticed for P3.2 (98.89%) and P2.2 (90.41%), indicating that the porphyrin derivatives might have relatively long half-life values after systemic administration. The highest values for steady-state volume of distribution were predicted for P3.1 (0.5420 L/kg). Furthermore, only P2.1 and P3.1 were predicted as permeable through the BBB. The highest values for total clearance were predicted for P3.1 (8.185 mL/min/kg) and P3.2 (7.396 mL/min/kg), while the other derivatives had comparable values. Nonetheless, none of the derivatives were predicted as substrates or inhibitors of renal transporters OCT1 and OCT2.

The predicted value of rat LD_50_ was 3066 mg/kg, suggesting that all compounds fall into the toxicity class V (class I—highest toxicity, class VI—lowest toxicity). The maximum tolerated dose after chronic administration in humans was predicted as 2.735 mg/kg/day for P2.1 and P2.2, and 2.742 mg/kg/day for P3.1, P3.2, and P4.2. All compounds were negative for predicted skin sensitization. Hepatotoxicity prediction was inconclusive for all porphyrin derivatives, although the ProTox-II algorithm estimated a 0.56 probability of hepatotoxicity for P2.1, P2.2, P3.1, P3.2, and 0.59 for P4.2. Unfortunately, all compounds were predicted as positive for nephrotoxicity and reproductive toxicity. Interestingly, only P3.1 was predicted as non-toxic to immune cells, with a 0.95 probability of being inactive, while the probability of posing immunotoxicity risks for the other derivatives ranged from 0.75 (P2.1) to 0.98 (P4.2). Moreover, only P3.1 and P4.2 were negative for carcinogenicity, while the results were inconclusive for the other compounds, with 0.5 and 0.51 probabilities of being carcinogenic. Furthermore, only P2.1 was predicted as potentially mutagenic, with a probability of 0.51. Predicted cytotoxicity was negative for all compounds, P3.1 showing the highest probability of lacking toxicity to healthy cells (0.74). Lastly, P3.1 was the only porphyrin derivative that lacked mitochondrial toxicity, according to the predictive models.

### 2.4. Cellular Uptake of the Investigated Porphyrins

Cellular uptake of the investigated porphyrins was evaluated on human skin-relevant cell lines: HaCaT keratinocytes, Hs27 fibroblasts, and SCL II squamous cell carcinoma. Mouse B16F10 melanoma cells were also investigated for comparison.

**Figure 10:**
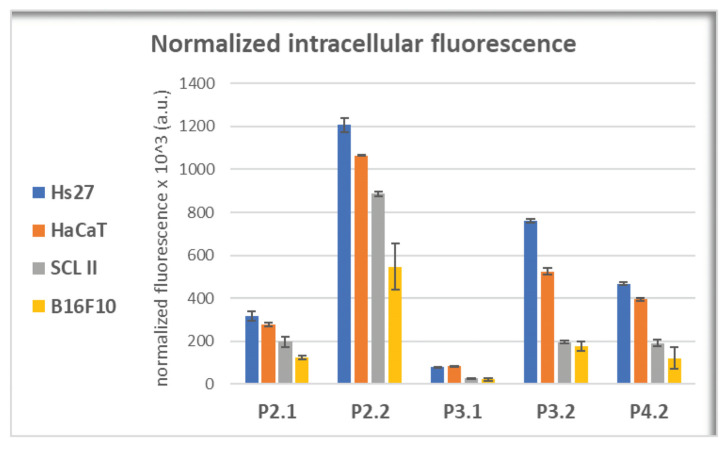
Intracellular fluorescence of porphyrins following 24 h incubation of porphyrins (10 μM) with skin-relevant cells (human Hs27 fibroblasts, human HaCaT keratinocytes, human SCL II squamous cell carcinoma, and murine B16F10 melanoma cells). Intracellular fluorescence was assessed by flow cytometry as geometric mean of the fluorescence signal normalized to the corresponding fluorescence emission of the porphyrin in PEG 200/PBS (1/1000) as solvent. Data are presented as mean value ± SD for 2–3 independent experiments in which all porphyrins were investigated simultaneously.

For assessing the cellular uptake of porphyrins, we took advantage of their fluorescent properties (Figure 4), and measured by flow cytometry the mean fluorescence of cells suspended in PBS. Cells were incubated in the previous 24 h with porphyrins at 10 μM concentration in culture medium. Considering that the investigated compounds had different intensities of the emission peak, the measured intracellular fluorescence was normalized to the corresponding maximal fluorescence intensity of each porphyrin, for having reliable data on cellular uptake, independent of the fluorescent properties of each porphyrin. Nevertheless, we did not take into account the potential decrease of the intracellular fluorescent signal due to the interaction of porphyrins with intracellular components. According to the flow cytometry data (Figure 10), P2.2 had the best uptake in all the investigated cell lines (*p* < 0.05), followed by P3.2, P4.2, and P2.1 (symmetrical compound). The lowest incorporation into cells was registered for the P3.1 compound, a result matched to the computational study which showed that P3.1 and P3.2 required high, positive transfer energies to permeate across the membrane core, higher energy values being also registered at the water–lipid interface. Although P3.2 was predicted through computational methods to have the lowest uptake (Figure 9), this was not confirmed by the in vitro study. For all the investigated porphyrins, the uptake was higher in normal fibroblasts and keratinocytes, while lower values were registered in SCL II and B16F10 tumor cells, with P2.2 having the best incorporation profile into cells, either non-malignant or malignant (Figure 10). Although the symmetrical P2.1 compound had the best fluorescent properties (Figure 4), its incorporation in cells was significantly lower than that of the asymmetric P2.2 compound in terms of intracellular fluorescence, probably due to its higher aggregability, as shown by the AFM measurements (Figure 5a).

Altogether, results indicate that, from the point of view of cellular uptake, the asymmetric P2.2 porphyrin is most suitable for incorporation in non-malignant or malignant skin cells, with best results in normal cells.

### 2.5. In Vitro Effects of Photodynamic Therapy on Investigated Porphyrins

The ability of the investigated porphyrins to kill cells by PDT was investigated in vitro on human skin-relevant cell lines: Hs27 fibroblasts, HaCaT keratinocytes, and SCL II squamous cell carcinoma cells. Mouse B16F10 melanoma cells were also investigated for comparison.

**Figure 11:**
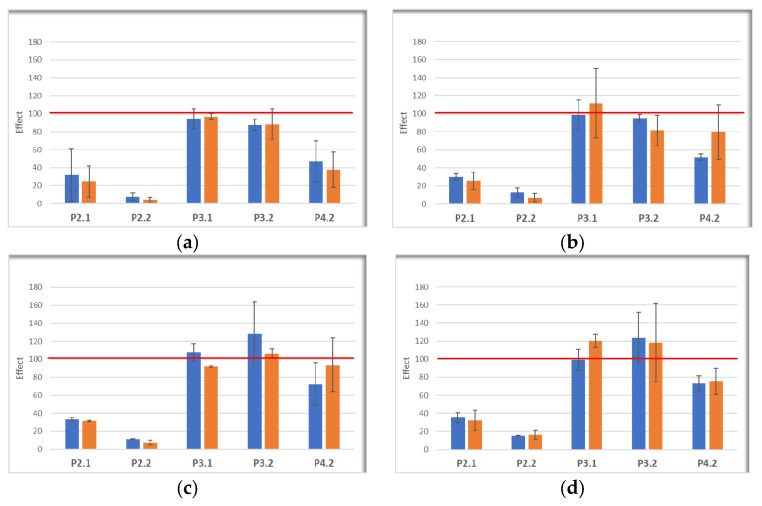
The MTS reduction by skin cells incubated with porphyrins (10 μM), and thereafter exposed to PDT (10 J/cm^2^, 50 mW/cm^2^). Measurements were performed at 24 h (blue) and 48 h (orange) after PDT. The effect of PDT on MTS reduction was calculated as: (cellular response in the presence of PDT)/(cellular response in the absence of PDT) × 100. Red line designates the 100% effect (no effect). Results are presented as mean effect ± SD for 2–3 independent experiments. (**a**) Human Hs27 skin fibroblasts; (**b**) Human HaCaT keratinocytes; (**c**) Human SCL II squamous cell carcinoma cells; (**d**) Murine B16F10 melanoma cells. Data are presented as mean value ± SD for 3 independent experiments in which all porphyrins were investigated simultaneously.

After incubating cells with porphyrins (10 μM) for 24 h, PDT was performed using laser light of 635 nm, at fluences previously communicated by us (10 J/cm^2^, 50 mW/cm^2^) [39]. After PDT, cells were cultivated for another 24 h or 48 h for establishing the impact of PDT at the level of MTS reduction, which provides information on the number of metabolically active cells, and of lactate dehydrogenase (LDH) release, which assesses the disturbance of the plasma membrane integrity due to cell death by necrosis [40]. The MTS reduction data (Figure 11) showed that P2.2 and, to a slightly lesser extent, the symmetrical P2.1 compound induced a drastic decrease in the number of metabolically active cells at 24 h, the effect being persistent also at 48 h after PDT. The effect of porphyrin P4.2 depended on the cell-type. Thus, P4.2 decreased the intensity of MTS reduction in Hs27 fibroblasts exposed to PDT, at levels similar to those elicited by PDT with the symmetrical P2.1 compound, and only modestly decreased the number of metabolically active HaCaT keratinocytes, SCL II squamous cell carcinoma cells, and B16F10 melanoma cells as compared to P2.1, especially at 24 h. Meanwhile, we did not register a consistent decrease in MTS reduction in any of the investigated cell lines exposed to PDT with P3.1 and P3.2, although P3.2 was well incorporated by the investigated cells, especially by fibroblasts and keratinocytes.

**Figure 12:**
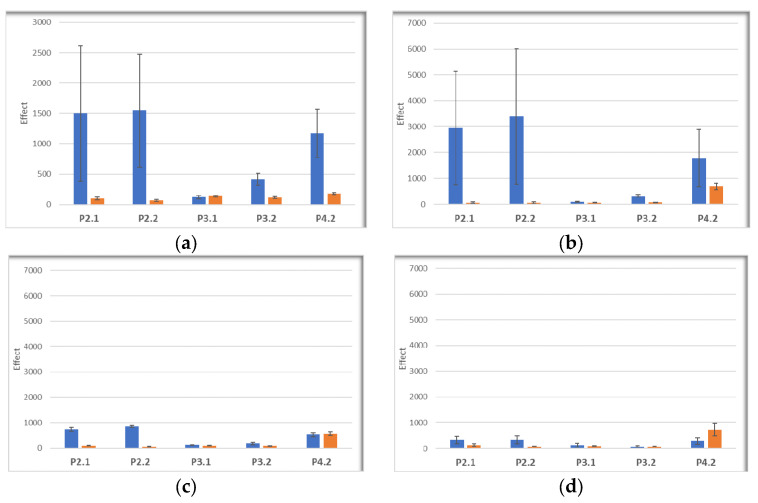
LDH release by cells incubated with porphyrins (10 μM), and thereafter exposed to PDT (10 J/cm^2^, 50 mW/cm^2^). Measurements were performed at 24 h (blue) and 48 h (orange) after PDT, in the same cellular samples in which MTS reduction was assessed (Figure 11). The effect of PDT on the LDH release was calculated as: (cellular response in the presence of PDT)/(cellular response in the absence of PDT) × 100. Results are presented as mean effect ± SD for 2–3 independent experiments. (**a**) Human Hs27 skin fibroblasts; (**b**) Human HaCaT keratinocytes; (**c**) Human SCL II squamous cell carcinoma cells; (**d**) Murine B16F10 melanoma cells. Data are presented as mean value ± SD for 3 independent experiments in which all porphyrins were investigated simultaneously.

For investigating cell death by necrosis following PDT, LDH release was measured in the same cellular samples in which MTS reduction was assessed. LDH release (effect > 500%) was registered within the first 24 h after PDT in Hs27 fibroblasts and HaCaT keratinocytes exposed to PDT with the P2.1, P2.2, and P4.2 compounds (Figure 12a,b), indicating that the decrease in metabolically active cells after PDT was partly due to rapid cell death by necrosis. The LDH release response was smaller in the case of tumor cells (Figure 12c,d), for which the effect of PDT on MTS reduction was also low (Figure 11). While an increased LDH release was confined to the first 24 h after PDT in the case of P2.1 and P2.2, cell death by necrosis continued to occur also at 48 h in the case of PDT with P4.2. Meanwhile, no significant increase in LDH release was registered when PDT was performed with the P3.1 and P3.2 compounds, for which MTS reduction also had nonsignificant changes (Figure 11).

Altogether, results point to the asymmetric P2.2 porphyrin as a valuable candidate for PDT in malignant skin disorders, with the amendment that normal skin around the tumor should be protected from PDT.

A limitation of our study is the lack of investigations regarding the concentration of intracellular ROS generated by PDT, singlet oxygen being the main produced cytotoxic oxidant following irradiation [41,42].

## 3. Materials and Methods

### 3.1. General Information Materials

Commercially available chemicals and solvents from Sigma-Aldrich (St. Louis, MO, USA) and Merck (Whitehouse Station, NJ, USA) were used.

### 3.2. Photophysical Characterization of Porphyrins

The investigated porphyrins were obtained according to the methods previously reported by us [27,28,29]. Because of their structural configurations, these compounds have demonstrated an excellent solubility in biologically friendly media, and long-term stability in PEG 200, a non-toxic and green pharmacologically accepted solvent [43]. The stock solutions of porphyrins (10 mM) were prepared in PEG 200, and were further diluted for experiments to 10 μM in PEG 200, PEG 200/PBS (1/1000), or in cell culture medium, depending on the type of experiment. For preventing uncontrolled photodegradation, porphyrin stocks in PEG 200 were kept in “dark” conditions at room temperature. Working solutions of porphyrins and cells loaded with porphyrins were manipulated in “dark” conditions as well, except when PDT was performed.

The spectral behavior of the porphyrinic structures was assessed by UV-Visible and fluorescence spectroscopy. The UV–Vis spectra of the porphyrins were registered with a Specord 200 spectrophotometer (Analytik Jena, Jena, Germany). Fluorescence spectra were registered using a steady-state Jasco FP 6500 spectrofluorometer (JASCO Co., Ltd., Kyoto, Japan) in 10 mm path length quartz cuvettes.

### 3.3. Evaluation of the Aggregation Statuss of Porphyrins

Atomic force microscopy (AFM) measurements were performed in non-contact mode as recommended for soft samples, using the XE-100 model from Park Systems (Suwon, Republic of Korea). All AFM images were scanned with NCHR sharp tips from Nanosensors™ (Neuchatel, Switzerland), having less than 8 nm tip radius, ~125 μm length, ~30 μm width, and ~42 N/m spring constant/~330 kHz resonance frequency. All porphyrins were prepared for AFM investigations as 10 μM solutions in PEG 200. A drop of the solution was deposited onto clean microscopic glass substrate (Heinz Herenz Medizinalbedarf GmbH, Hamburg, Germany), and was let to dry at room temperature, in the dark. AFM images were registered over several scanning areas of (10 × 10) μm^2^ and (2 × 2) μm^2^, and were further processed with the Image Processing Program, XEI—v.1.8.0, developed by Park Systems (Suwon, Republic of Korea) for display purpose (1st order tilt correction). To have a better view of the surface morphology, beside classical colored (mono-color brown gradient), the AFM images were presented in the so-called “enhanced color” (EC), which uses the change of a pixel relative to its neighbors. Below the 2D EC AFM images, an arbitrary line (one-line scans collected along the X-scan direction) was presented for each surface, showing in detail the surface profile of each investigated surface. Further on, the AFM images were processed with Scanning Probe Image Processor software SPIP™ v. 4.6.0.0 (Lyngby, Denmark) [44] for surface amplitude parameters evaluation, as root mean square (RMS)—(Rq) and average (R_a_) roughness, as well as the peak-to-peak height (R_pv_), which quantitatively describes the surface corrugation degree. The root-mean square roughness (Rq) represents the standard deviation of the height value; the average roughness (Ra) is the area between the roughness and its mean, while the peak-to-valley parameter (Rpv) is the height difference between the lowest and highest points in the scanned area. The textural properties were evaluated by mean fractal dimension (MFD) and texture direction index (S_tdi_). Skewness (Rsk) and kurtosis (Rku) were associated to the distribution of the heights, with Rku describing the randomness of heights profile, and Rsk describing the asymmetry of the height distribution. The self-similarity, which reflects the property that a part of the surface is similar to the whole surface, was estimated by calculating the mean fractal dimensions (MFD), while the texture/isotropy was assessed by Stdi parameters (related to the weight of the dominant direction).

### 3.4. Computational Studies

#### 3.4.1. Permeability across Cell Membrane Prediction

The ability of the investigated porphyrin derivatives to diffuse across the cell membrane was predicted using the PerMM (Permeability of Molecules across Membranes) web-server (University of Michigan, Ann Arbor, MI, USA) [45]. PerMM is a thermodynamics-based method that simulates the translocation of chemical compounds across a lipid bilayer consisting of dioleoyl phosphatidylcholine. Three-dimensional structures of the investigated compounds were generated using DataWarrior [46], and were thereafter energetically minimized using the MMFF94s+ force field. Simulations were performed at 298 K and physiological pH (7.4). The deionization energy was not considered, since the studied compounds protonate in acidic medium at pH values lower than 6. Using PerMM, cell membrane binding affinity (ΔG, kcal/mol) and permeability coefficients (log Perm) were calculated, and transfer energy profiles were generated as a function of distance from the membrane center.

#### 3.4.2. ADMET Profile Prediction

The absorption, distribution, metabolism, excretion, and toxicity (ADMET) properties of the investigated porphyrin derivatives were predicted using 3 web-servers: admeSAR [47], pkCSM [48], and ProTox-II [49]. If prediction results were different among the web tools for the same compound, the predicted activity class was labeled as “inconclusive”.

### 3.5. Cellular Studies

#### 3.5.1. Cells

Porphyrins were investigated in cellular systems using standardized cell lines purchased from ATCC (Manassas, VA, USA)—Hs27 fibroblasts from human foreskin, and B10F10 murine melanoma cells, and from CLS Cell Lines Service GmbH (Eppelheim, Germany)—HaCaT spontaneously immortalized keratinocytes from adult human skin, and SCL II squamous cell carcinoma cell lines from face skin.

Cells were maintained and multiplied in DMEM-F12 culture medium with GlutaMAX (Gibco, Waltham, MA, USA), supplemented with 10% fetal bovine serum (FBS, Sigma, Saint Louis, MO, USA), further referred to as complete culture medium, at 37 °C in 5% CO_2_ atmosphere. Cells were periodically detached with 0.05%/0.02% (*w*/*v*) Trypsin-EDTA (Biochrom, Cambridge, UK), split, and recultivated. Cells were counted by optical microscopy in a Burker-Turk counting chamber using Trypan blue as dead cells stain. Cell cultures with a viability >95% were used for experiments.

#### 3.5.2. Loading of Cells with Porphyrins

Cells, seeded in 24 well plates or in 35 mm Petri dishes, were cultivated for 24 h in complete culture medium, at 37 °C, in 5% CO_2_ atmosphere, for allowing their adherence. For porphyrins loading, the complete culture medium was replaced with DMEM-F12 culture medium with GlutaMAX (Gibco, Waltham, MA, USA), supplemented with 2% FBS (Sigma, Saint Louis, MO, USA) and 10 μM porphyrins, and cells were cultivated for another 24 h.

#### 3.5.3. Evaluation of Porphyrin Uptake by Cells

For measuring the uptake of porphyrins into the normal and tumor cells, the protocol described at point 3.5.2 was performed in 24 well plates. A cell culture without porphyrins was used as control for measuring cellular autofluorescence. At the end of the protocol, cells were detached with 0.05%/0.02% (*w*/*v*) Trypsin-EDTA (Biochrom, Cambridge, UK), washed by centrifugation in PBS, and fixed for 15 min, at room temperature in the dark with FluoroFix™ Buffer (BioLegend, Sandiego, CA, USA). Finally, after removing the fixative by centrifugation, cells were suspended in PBS and subjected to investigations by flow cytometry on a BD FACSCanto II cytometer with BD FACSDiva 6.1 software (Becton Dickinson, Fraklin Lake, NJ, USA), using the 488 nm laser. Fluorescence data from at least 5.000 events were collected, and the geometric mean of the fluorescent signal distribution in the FL3 channel was computed automatically by the software in arbitrary units. As the investigated porphyrins had different emission maxima at 635 nm (excitation at 410 nm), the intracellular fluorescence signal was normalized to the maximum fluorescence emitted by each porphyrin. Data from independent experiments were processed as mean value ± standard deviation (SD).

#### 3.5.4. In Vitro PDT

After loading of cells with porphyrins in 35 mm Petri dishes (see Section 3.5.2), the porphyrin-containing culture medium was discarded, and 1 mL Hank’s balanced salt solution supplemented with 2% FBS was added. Cells were immediately subjected to PDT, while control cultures, that were not treated with porphyrins and were not subjected to PDT, were kept in the “dark” at room temperature. PDT was performed in test samples at room temperature, using a ML6600 instrument (Modulight, Tampere, Finland) equipped with a 635 nm laser, illumination chamber for 35 mm Petri dishes, and software control of temperature and PDT parameters (light fluence, irradiance, power, and time). The applied PDT parameters were 10 J/cm^2^ fluence and 50 mW/cm^2^ irradiance.

#### 3.5.5. Post-PDT Cellular Investigations

Immediately after PDT, the PDT culture medium was removed and was replaced with 1.5 mL complete culture medium. Both test samples and controls were further cultivated for 24 h in 5% CO_2_ atmosphere at 37 °C. Aliquots of cell culture supernatant were harvested for measuring the LDH release. Cells were thereafter detached with 0.05%/0.02% (*w*/*v*) Trypsin-EDTA (Biochrom, Cambridge, UK), suspended in 5 mL complete culture medium for trypsin annihilation, centrifuged, and suspended in complete culture medium. Cells were counted (see point Section 3.5.1) and distributed in triplicates in 96 well plates for measuring MTS reduction at 24 h after PDT, and MTS reduction and LDH release at 48 h post-PDT, as will be described below.

MTS reduction, which provides information on the number of metabolically active cells [50], was assessed using the colorimetric CellTiter 96^®^ AQueous One Solution Cell Proliferation Assay (MTS) from Promega (Madison, WI, USA), according to the manufacturer’s procedure. Briefly, 20 μL of the kit reagent was added to each well, and samples were cultivated 2 h at 37 °C. Finally, the optical density at 490 nm was measured against the 620 nm wavelength reference, using a Sunrise Tecan microplate reader equipped with universal reader control and Magellan data analysis software v6.0 (Tecan, Männedorf, Switzerland). Data were expressed as optical density (OD) of each cellular sample, corrected for background by subtracting the mean optical density of complete cell culture medium. The mean and standard error of the mean were calculated for sample triplicates. Data were finally processed as PDT effect calculated by dividing the mean OD of the PDT-treated sample by the mean OD value of the control sample (not loaded with porphyrins, and not treated by PDT). The MTS reduction evidencing, therefore, the impact of PDT on cellular viability and proliferation.

LDH release was used for evaluating membrane integrity of PDT-treated and non-treated cells. The method provides information on cell death through necrosis/necroptosis [40].

Cell-free supernatants harvested at 24 h or 48 h post-PDT were tested for LDH release.

LDH release was measured using the CytoTox 96^®^ Non-Radioactive Cytotoxicity Assay (Promega, Madison, WI, USA). According to the manufacturer’s procedure, 50 μL of cell-free supernatant and 50 μL of LDH substrate were incubated for 30 min in the dark, at room temperature. The reaction was interrupted by the addition of 50 μL stop solution. The OD of samples in triplicate was measured at 490 nm using a Sunrise Tecan microplate reader equipped with universal reader control and Magellan data analysis software (Tecan, Männedorf, Switzerland). Data were expressed as optical density (OD) of each cellular sample, corrected for background by subtracting the mean OD of complete cell culture medium without cells. The mean and standard error of the mean were calculated for sample triplicates. Data were finally processed as PDT effect calculated by dividing the mean OD of the PDT-treated sample by the mean OD value of the corresponding control sample (not loaded with porphyrins, and not treated by PDT).

Results from triplicate samples were processed as mean value ± standard error of the mean, while results from independent experiments were presented as mean value ± SD. Comparison between the investigated compounds was performed in Excel, using the *t*-Test: Paired Two Sample for Means, considering that all porphyrins were tested in parallel in each experiment.

## 4. Conclusions

All samples were found to be isotropic and self-similar, with slightly different degrees of aggregability, had a relatively low predicted toxicity (class V), and a predicted long half-life after systemic administration. The in vitro study performed on non-malignant and malignant skin-relevant cells highlighted that the asymmetric P2.2 porphyrin qualified among the five investigated porphyrins to be a promising photosensitizer candidate for PDT in skin disorders. P2.2 was shown to accumulate well within cells, although it had a certain degree of aggregation in PEG 200, and induced by PDT a massive decrease in the number of metabolically active skin cells, partly due to cell death by necrosis within the first 24 h post-PDT. P2.2 had in this respect a better behavior than the symmetric P.2.1 compound which, albeit exhibiting the best fluorescent properties, had a higher degree of aggregation. P2.2 was also superior to the related asymmetric compound P4.2. The strong action of P2.2-mediated PDT on normal skin cells might be an important drawback for further development of this compound, along with some ADMET predictions highlighting the risk of nephron- and reproductive toxicities for all the investigated porphyrins. The P3.1 and P3.2 compounds had a predicted high value of total clearance, and were not able to accumulate well in skin cells, although they exhibited a lower degree of aggregability than P2.2, and did not elicit significant PDT in vitro consequently. Therefore, we propose compound P2.2 for future investigations, to assess the effects on intracellular ROS production and regulation of relevant genes following PDT, to further develop novel, effective photosensitizers for PDT in skin disorders.

## 5. Patents

a. Patent No. 132752 B1: Rica Boscencu, Gina Manda, Radu Petre Socoteanu, Mihail Eugen Hinescu, Ionela Victoria Neagoe, Laura Olariu, Brandusa Dumitriu, *Porphyrin derivative for theranostic use*, published in RO-BOPI, 11 from 29 November 2023.

b. Patent application 201900799: Rica Boscencu, Gina Manda, Laura Olariu, Ionela Victoria Neagoe, Radu Petre Socoteanu, Mihail Eugen Hinescu, Luis Filipe Vieira Ferreira, Antonio Cuadrado, Huveida Basaga, *Tetrapyrrolic derivative for antitumor photodynamic therapy and obtaining process*, published in RO-BOPI, 9 from 30 September 2020.

## Figures and Tables

**Table 1:** Photophysical properties of porphyrins (10 μM final concentration, λ_ex_ = 410 nm) in PEG 200, and in PEG 200/PBS (1/1000).

Solvent	Absorption λ_max_ (nm) [lg ε] (L mol^−1^ cm^−1^)					Emission λmax(nm) [F.I.] (a.u.)
	Soret Band	Qy(1,0)	Qy(0,0)	Qx(1,0)	Qx(0,0)	
*5,10,15,20-tetrakis-(4-acetoxy-3-methoxyphenyl) porphyrin*						
**PEG 200**	402 [5.326]	495 [4.097]	531 [3.851]	569 [3.552]	626 [3.501]	652 [22.560]
**PEG 200/PBS**	405 [5.189]	499 [4.330]	534 [4.086]	571 [3.900]	628 [3.786]	654 [21.920]
*5-(4-hydroxy-3-methoxyphenyl)-10,15,20-tris-(4-acetoxy-3-methoxyphenyl) porphyrin*						
**PEG 200**	405 [5.383]	498 [4.136]	535 [3.947]	572 [3.556]	629 [3.585]	650 [18.395]
**PEG 200/PBS**	404 [5.332]	498 [4.247]	535 [4.094]	571 [3.902]	621 [3.890]	656 [9.523]
*5,10,15,20-tetrakis-(4-carboxymethylphenyl) porphyrin*						
**PEG 200**	400 [5.360]	494 [4.071]	527 [3.736]	568 [3.448]	625 [3.235]	650 [21.076]
**PEG 200/PBS**	406 [5.062]	498 [4.205]	531 [3.930]	571 [3.755]	628 [3.552]	656 [19.804]
*5-(2-hydroxy-3-methoxyphenyl)-10,15,20-tris-(4-carboxymethylphenyl) porphyrin*						
**PEG 200**	400 [5.161]	495 [3.942]	526 [3.613]	568 [3.310]	626 [3.101]	656 [13.850]
**PEG 200/PBS**	405 [4.813]	499 [3.983]	530 [3.683]	571 [3.476]	629 [3.253]	657 [5.206]
*5-(2,4-dihydroxyphenyl)-10,15,20-tris-(4-acetoxy-3-methoxyphenyl) porphyrin*						
**PEG 200**	402 [5.269]	496 [3.990]	532 [3.710]	570 [3.322]	626 [3.085]	652 [17.291]
**PEG 200/PBS**	407 [5.010]	498 [4.138]	533 [3.885]	571 [3.670]	627 [3.467]	655 [9.472]

**Table 2:** Predicted values for cell membrane binding affinities (ΔG) and permeability coefficients (logPerm).

Compound	ΔG (kcal/mol)	logPerm
2.1	−6.29	0.65
P2.2	−7.15	1.28
P3.1	−5.86	−1.50
P3.2	−5.49	−3.08
P4.2	−6.41	0.62

**Table 3:** Predicted ADME parameters for the investigated porphyrin derivatives.

Parameter	P2.1	P2.2	P3.1	P3.2	P4.2
Intestinal absorption	yes	yes	yes	yes	yes
Oral bioavailability	low	low	low	high	low
P-glycoprotein substrate	inc.	inc.	inc.	no	yes
P-glycoprotein inhibitor	yes	yes	yes	yes	yes
OATP subtypes inhibition	yes	yes	yes	yes	yes
Skin permeability (log Kp, cm/h)	−2.735	−2.735	−2.735	−2.735	−2.735
Subcellular localization	mitochondria	mitochondria	mitochondria	mitochondria	mitochondria
CYP isoforms inhibitor	inc.	inc.	inc.	inc.	inc.
Plasma protein binding	0.8118	0.9041	0.8745	0.9889	0.8754
VDss (L/kg)	0.4446	0.4217	0.5420	0.4742	0.4305
BBB permeability	yes	no	yes	no	no
Total clearance (mL/min/kg)	4.656	4.285	8.185	7.396	4.581
OCT1/2 substrate/inhibitor	no	no	no	no	no

inc.—inconclusive; OATP—organic anion transporting polypeptide; VDss—steady-state volume of distribution; BBB—blood brain barrier; OCT—organic cation transporter.

**Table 4:** Predicted toxicity of the investigated porphyrin derivatives.

Parameter	P2.1	P2.2	P3.1	P3.2	P4.2
Rat LD_50_ (mg/kg)	3066	3066	3066	3066	3066
Human max. tolerated dose (mg/kg/day)	2.735	2.735	2.742	2.742	2.742
Skin sensitization	no	no	no	no	no
Hepatotoxicity	inc.	inc.	inc.	inc.	inc.
Nephrotoxicity	yes	yes	yes	yes	yes
Reproductive toxicity	yes	yes	yes	yes	yes
Immunotoxicity	yes	yes	no	yes	yes
Carcinogenicity	inc.	inc.	no	inc.	no
Mutagenicity	yes	no	no	no	no
Cytotoxicity	no	no	no	no	no
Mitochondrial toxicity	yes	yes	no	yes	yes

LD_50_—median lethal dose, inc.—inconclusive.

## Data Availability

Data are contained within the article.
